# Malformation of the brainstem accompanied by cortical dysplasia

**DOI:** 10.1590/0100-3984.2016.0216

**Published:** 2018

**Authors:** Sergio Eiji Ono, Débora Brighente Bertholdo, Gustavo Rengel dos Santos, Arnolfo de Carvalho Neto

**Affiliations:** 1 Clínica Diagnóstico Avançado por Imagem - DAPI, Curitiba, PR, Brazil; 2 Hospital de Clínicas da Universidade Federal do Paraná (UFPR), Curitiba, PR, Brazil

**Figure 1:**
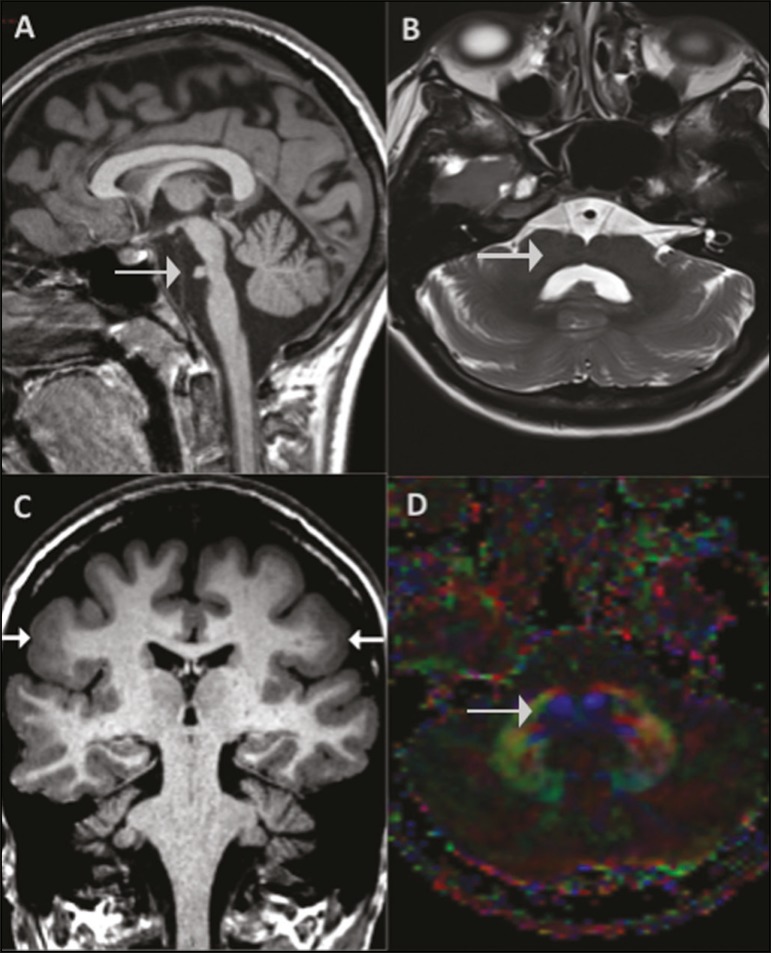
**A:** Sagittal T1-weighted image depicting a short pons (arrow). **B:** Axial T2-weighted image at the pons-medulla junction showing a ventral cleft (arrow). **C:** Coronal T1-weighted image showing cortical dysplasia (arrows) with a thickened cortex. **D:** Axial fractional anisotropy color map showing the absence of transverse pontine fibers and of the medial lemniscus (arrow).

Dear Editor,

We present the case of a 20-year-old woman referred for investigation of epilepsy. A magnetic resonance imaging (MRI) study (Figure 1) showed bilateral areas of focal cortical dysplasia (FCD) along the perisylvian cortex, together with a brainstem malformation characterized by a ventral cleft at the pons-medulla junction. Diffusion tensor imaging (DTI) revealed the absence of transverse pontine fibers and of the medial lemniscus.

Midbrain-hindbrain (MBHB) malformations include a large group of posterior fossa malformations, with different mechanisms and genetic components involved. The clinical findings are nonspecific, varying from hypotonia to seizures and lack of developmental progress^(1)^. A recent classification of MBHB malformations proposed by Barkovich et al.^(2)^ is based mainly on embryology and genetics^(3)^. According to that classification system, the ventral cleft seen in our case suggests a regional (group III) developmental defect. Predominantly brainstem malformations may be better evaluated in MRI with threedimensional, heavily T2-weighted, steady-state sequences, which allow adequate visualization of the cranial nerve in the basal cisterns. DTI of the brainstem may also be helpful and shows promise for further delineating axonal path disorders of the brainstem in the absence of obvious structural defects^(1)^. Although MBHB malformations can occur in isolation, many of them are accompanied by other malformations, particularly supratentorial malformations, which tend to have a significant effect on the prognosis of these patients. Severe hypoplasia of the pons and medulla with a dorsal cleft and absence of the fascial colliculus can occur in a recently described syndrome-horizontal gaze palsy with progressive scoliosis-which is a rare autosomal recessive disease, characterized by congenital absence of conjugate horizontal eye movements, preservation of vertical gaze, preservation of convergence, and progressive scoliosis, that develops in pediatric patients. The progressive scoliosis is probably secondary to neurological deficits that impair proprioceptive inputs^(4)^.

Our patient had FCD, which is a major cause of epilepsy in children and adults^(3)^. FCD type II, also known as FCD with the transmantle sign or Taylor-type dysplasia, is classified as a category I malformation of cortical development (MCD), because it involves abnormal neuronal proliferation. The other MCD categories include abnormalities in neuronal migration (category II-e.g., periventricular nodular heterotopia) and abnormal late migration/cortical organization (category III-e.g., FCD type I and polymicrogyria)^(5)^.

In a study of 220 patients with MCD and epilepsy, Kuchukhidze et al.^(5)^ analyzed the combination of MBHB malformations and FCD. The authors identified MBHB malformations in 17% of the patients and found that the malformations were more commonly linked to late migration/cortical organization disorders; only one patient was found to have FCD type II. The cases of MBHB malformations were associated with more extensive MCD lesions, as well as with a poor clinical profile (earlier age at seizure onset, neurologic deficits, learning disability, and developmental delay), although no differences were found in the response to antiepileptic treatment. Nearly 25% of the patients with MBHB malformations had FCD type I, which was not detected in MRI studies and was identified only through pathologic examination of a surgical specimen.

Studies of MBHB malformations have improved with advances in neuroimaging, molecular biology, and molecular genetics, thus increasing understanding of developmental disorders related to such malformations. Functional MRI techniques can also contribute to a better description and understanding of these diseases.
